# Redefining Role of 5-Fluorouracil and Exploring the Impact of Taxanes and Cisplatin in Locally Advanced and Recurrent Carcinoma Cervix in Concurrent Setting With Radiotherapy: A Literature Review

**DOI:** 10.7759/cureus.11645

**Published:** 2020-11-23

**Authors:** Jasmeet Kaur, Sapna Marcus, Shreya Garg, Anju Kansal

**Affiliations:** 1 Internal Medicine, Saint Joseph Mercy Oakland Hospital, Pontiac, USA; 2 Radiation Oncology, All India Institute of Medical Sciences, Bathinda, IND; 3 Obstetrics and Gynaecology, Guru Gobind Singh Medical College and Hospital, Faridkot, IND; 4 Radiation Oncology, Civil Hospital Bathinda, Bathinda, IND

**Keywords:** concomitant chemo-radiation therapy, carcinoma cervix, cisplatin, 5-fu, paclitaxel, chemotherapy agents, radio sensitization

## Abstract

Cervical carcinoma is the fourth most frequent cancer among women worldwide while it is common in rural India. The irony of the situation is that it continues to present in a locally advanced stage with bulky disease posing a significant challenge to the current treatment modalities despite various screening programs. Concurrent chemoradiotherapy is the mainstay of treatment for locally advanced carcinoma cervix. However, the appropriate dosing schedules, along with the salutation of the chemotherapeutic agent, remain a matter of debate to date. The use of chemotherapy in the neoadjuvant and adjuvant setting promises to improve progression-free survival and overall survival. The article aims to review various chemotherapy and their regimens in the treatment of carcinoma of the cervix.

## Introduction and background

Cervical cancer is the fourth most common cancer and the fourth leading cause of cancer-related mortality worldwide in women [1]. “There were approximate 570,000 cases of cervical cancer and 311,000 deaths due to cervical cancer worldwide in 2018” [2,3]. “The worldwide age-related estimated Incidence of cervical cancer as of 2018 was 13.1 per 100,000 women” [2,3]. One-third of total cervical cancer patients worldwide are contributed by China and India together with “106,000 cases in China and 97,000 cases in India” [2,3]. The developing countries account for around ninety percent of deaths due to cervical cancer. India accounted for 20% of cervical cancer deaths (60,000) [2,3]. The human papillomavirus (HPV) can be identified in more than 90% of cervical cancer, and cervical cancer infection is considered the most common cause of most cervical cancer [3,4]. Other risk factors are young age at first intercourse, smoking, lack of circumcision in the male partner. Women who have intercourse at a young age are more prone to cervical cancer [5]. The cervical Incidence of cancer has been reduced worldwide, with frequent screening and HPV vaccination [5]. The screening and HPV vaccination lacks in developing countries [6]. The treatment for early-stage carcinoma cervix is surgery and chemoradiotherapy, while for advanced stage, chemoradiation therapy is the mainstay of treatment. The purpose of the article is to review various chemotherapy and their regimens in the treatment of cervical cancer with particular emphasis on the redefined role of 5-fluorouracil (5-FU), cisplatin and the impact of taxanes in carcinoma cervix in a concurrent setting with radiotherapy. 

## Review

Pathophysiology in carcinoma uterine cervix

Invasive cervical Carcinoma comprises approximately 85% squamous cell varieties, 15% adenocarcinoma and other histopathologic types [7]. The clinical prognosis for early cervical cancer is relatively good, while for advanced cervical cancer, the prognosis is poor with a high treatment failure rate [7]. The primary cause of treatment failure is the presence of a large bulky tumor with central necrosis and hypoxic area [8]. The persistent pelvic disease and recurrent disease develop into malignant clones resistant to chemotherapy, and radiation therapy leads to treatment failure [8]. 

Tumor microenvironment and hypoxia

The tumor microenvironment in cervical cancer has an essential role in tumor progression and invasion [9]. Vaupel et al., in their study on “blood flow, oxygen, nutrient supply and metabolic microenvironment of human tumors “demonstrates that in normal tissue, oxygen flow depends on the homeostatic equilibrium between oxygen consumption and oxygen delivery by the tissue. [10]. A Malthusian principle is applied to the fast-growing tumor, which is observed in an experimental tumor characterized by a rapid increase in tumor cell population consuming oxygen compared to the expansion of the functional microvasculature supplying oxygen to these structures [10]. Therefore, tissue hypoxemia can result from either impaired oxygen diffusion or perfusion due to a lack of vasculature [10]. The hypoxia and reoxygenation to the tumor tissue enhance tumor progression, local invasion, metastasis, and tumor resistance to the various treatment as observed in vitro and vivo preclinical studies [11]. Hypoxic cervical carcinoma has a poor outcome and significant cause of residual and relapse [12].

Standard treatment for locally advanced carcinoma cervix (Stage 2B to 4A)

The standard treatment for locally advanced carcinoma cervix (LACC) as per National Comprehensive Cancer Network (NCCN) guidelines is concurrent chemoradiation therapy, which further depends on the status of par-aortic lymph nodes involvement [13]. For patients without adenopathy, the standard treatment is radiation therapy (RT) with concurrent platinum-containing chemotherapy along with brachytherapy. The patients with para-aortic lymphadenopathy, the RT is provided to the involved nodes [13]. 

Standard treatment for recurrent carcinoma cervix

The treatment for recurrent carcinoma cervix as per NCCN guidelines depends on the prior RT status. Patients diagnosed at early stages and have not received RT can be candidates for either surgical resection or consider individualized RT with or without chemotherapy and brachytherapy [13]. 

Standard treatment for metastatic carcinoma cervix

Patients who presented with metastatic carcinoma cervix or the disease has progressed can be assessed for radiation treatment. If radiation treatment is not feasible, depending on the patients' performance status can be considered for systemic chemotherapy. The preferred regimens are cisplatin, carboplatin and paclitaxel-based chemotherapy [13].

Chemoradiation therapy in carcinoma cervix

Concurrent chemoradiation therapy is the most common approach for the treatment of locally advanced cervical cancer [13]. The tumor size limits the treatment with radiation therapy as it requires broad tumor volume coverage and radiation dose, which might involve normal surrounding tissue that exceeds their tolerance to radiation providing early and late radiation toxicity [14]. Radiation therapy with a low dose associated with high treatment failure and poor outcome [14]. The delicate balance between radiation treatment to the tumor and sparing normal tissue from radiation toxicity is difficult to maintain in many patients [14]. Radiotherapy alone, including external beam radiotherapy and intracavitary brachytherapy, has not shown promising results. A randomized control trials by Keys et al., Morris et al., and Whitney et al. have shown that with the use of concurrent chemotherapy with radiation (CTRT) improved the treatment outcome in patients with LACC in terms of tumor response and radiation toxicity [15-17]. 

Green et al. demonstrated in their study on “Concomitant chemotherapy and radiotherapy for cancer of the uterine cervix” demonstrated that concomitant chemoradiation the group shows improve overall survival and progression-free survival with tolerable systemic toxicity of chemotherapy and enhancing radiosensitization [18]. 

Five landmark randomized trial Keys et al., Whitney et al., Rose et al., Morris et al., Peters et al. established the modern treatment with a combination of concurrent chemotherapy with EBRT and intracavitary brachytherapy as the standard of care in the treatment of carcinoma cervix [15,16,17,19]. The National Cancer Institute of National Institute of Health (NIH) in 1999 issued an announcement based on these trials to strongly consider concurrent Cisplatin with radiation therapy in cervical cancer patients [20]. The standard first-line concurrent chemotherapy as per NCCN guidelines are radiosensitizing cisplatin-based chemotherapy in the management of advanced cervical cancer [13,21].

Role of cisplatin as chemotherapeutic agent in chemoradiation 

Cisplatin is a platinum analog chemotherapeutic drug. Barnett Rosenberg et al. in the 1960s discovered the cell-killing potential and antitumor activity of platinum compounds cisplatin developed in 1970 [22]. Cisplatin inhibits DNA synthesis by binding to DNA forming an adduct which inhibits DNA transcription promotes cell death in actively dividing cells such as tumor cell promoting antitumor activity [22,23]. Cisplatin binds preferentially to the adenine and guanine at the N7 position of DNA. The interaction at these two different sites on DNA produces DNA adducts and damages the replication and repair process leading to cell [24].

Evidence of cisplatin as radiosensitizer agent in treatment of carcinoma uterine cervix

Cisplatin is a radiosensitizing agent that enhances tumor cell killing in the presence of radiation therapy [20]. Radiation-induced cellular injury, including DNA damage repair, is inhibited in the presence of cisplatin, which induces further damage and promotes tumor cell killing [25]. Cisplatin enhances radiation effects in radioresistant hypoxic cells [25]. A clinical trial by GOG125 compared concurrent chemoradiation therapy with weekly cisplatin versus standard three-weekly cisplatin and 5-FU showed no significant difference in the outcome but reduced toxicity in cisplatin alone group [26]. This was a randomized control comparative study, on the patients in stage IIB, IIB and IVA cervical cancer. The patients received EBRT with concurrent weekly cisplatin compared with a protracted infusion of 5-fluorouracil followed by intra-cavitary brachytherapy. The outcome of this study did not demonstrate statistically difference in progression-free survival in 5-FU arm compared with weekly cisplatin [26].

In a phase III study comparing “radical radiotherapy with and without chemotherapy in patients with advanced squamous cell cancer of the cervix with pelvic lymph node involvement” showed no improvement in progression-free survival and mortality benefits of weekly cisplatin with concurrent RT over RT alone [27]. In a clinical trial by the “National Cancer Institute of Canada” on concurrent chemoradiation with single-agent cisplatin did not meet the primary endpoint in terms of disease-free survival between two groups [28]. In a study by Kim et al. in “locally advanced cervical cancer compared monthly fluorouracil and cisplatin versus cisplatin alone concurrent with external beam radiotherapy, and high dose rate brachytherapy” showed no difference in tumor response and overall survival but there is improvement in treatment compliance seen in patients with cisplatin alone group [29]. 

Role of 5-FU

The 5-FU is a halogenated pyrimidine that analog belongs to the antimetabolite chemotherapy class [30]. It is a cell cycle-specific chemotherapeutic agent with activity. In S-phase, 5-FU enters the cell by a facilitated uracil transport system and requires activation to cytotoxic nucleotide form [30]. It mainly acts by three mechanisms includes inhibition of thymidylate synthase, incorporation into DNA and RNA impairing their function and damages the cell cycle promotes cellular killing [30]. 

 Evidence of 5-FU as radiosensitizer agent in the treatment of carcinoma uterine cervix

The 5-FU activity as radiosensitizer is demonstrated by its selectively activity during S-phase of cell cycle [30]. It inhibits cell in the S phase of the cell cycle and inhibit the repair of radiation-induced DNA and RNA damage leading to permanent damage and cell death [30]. In a randomized study by Christie et al. showed that Concurrent chemotherapy, mitomycin C, and 5-FU together with conventional RT showed an improved DFS rate when compared with conventional RT alone in patients with locally advanced carcinoma of the cervix [31]. The role of 5-FU as a radiosensitizer is well established in carcinoma uterine cervix and has been evaluated in many randomized trials. Comparing radiation therapy with 5-FU with radiation therapy alone, Thomas et al., 1998 demonstrated a benefit in survival for patients [28]. The role of concurrent RT with cisplatin and 5-FU has also been studied in comparison with other chemotherapeutic agents such hydroxyurea, showed superior results in cisplatin arm [32]. However, the combination of 5-FU with cisplatin is more toxic.

Role of paclitaxel

Paclitaxel is a cell cycle-specific taxane analogue [33]. Schiff et al., 1979 defined the mechanism of action of paclitaxel. Paclitaxel activity is more commonly observed in the mitotic (M) phase of the cell cycle. It acts on the microtubule during spindle formation in mitotic phase leading to tubulin polymerization and inhibit spindle formation and cell division [34].

Evidence of paclitaxel as radiosensitizer agent in the treatment of carcinoma uterine cervix

Paclitaxel is cell cycle phase selective antitumor agent in G2 and M phase [35]. The G2 and M phase is the most radiosensitive phase to inhibit cell cycle during radiation treatment as the cell is highly active during these [35]. Preclinical studies in mouse model shown that paclitaxel enhances the effect of radiation treatment in human cervical cancer cell lines [35]. Moore et al., in their study, compare radiotherapy with concurrent cisplatin vs concurrent cisplatin and paclitaxel in patients with advanced cervical cancer demonstrated that weekly paclitaxel shows added clinical advantage [36]. 

Challenges in the treatment of locally advanced carcinoma cervix 

The major challenge in the treatment of cervical cancer is treatment failure with a high rate of recurrence and distant metastasis [19,27]. The current standard cisplatin-based approach is not effective in all patients. Future research should explore a new radiosensitizers agent and different chemotherapy regimens with radiotherapy including different combinations of cisplatin and 5-FU to enhance radiation effect and limit treatment failure in cervix cancer. A study comparing three weekly cisplatin and biweekly 5-FU versus weekly paclitaxel with concurrent radiotherapy showed no statistically significant difference between progression-free survival and overall survival [37].

## Conclusions

The prevalence of advanced cervical cancer has immensely reduced in the developed world by introducing cervical cancer screening. However, because of the lack of the screening process's lack of adoption due to limited screening, its prevalence is still at a peak in the developing world. Chemoradiation therapy remains the mainstay of treatment. There is a need to explore the new chemoradiation therapy regimens to prevent disease progression, recurrence and metastasis. The available cisplatin-based chemoradiation is the standard treatment therapy for advanced cervical cancer. There is a higher failure rate in bulky disease and para-aortic positive lymph node cervical cancer, and there is a need to investigate new treatment regimens in these patients. 
